# Impact of the leptin receptor gene on pig performance and quality traits

**DOI:** 10.1038/s41598-024-61509-1

**Published:** 2024-05-09

**Authors:** Rafael Suárez-Mesa, Roger Ros-Freixedes, Ramona N. Pena, Josep Reixach, Joan Estany

**Affiliations:** 1 Department of Animal Science, University of Lleida – Agrotecnio-CERCA Center, 191 Rovira Roure, 25198 Lleida, Catalonia Spain; 2 Selección Batallé S.A., 17421 Riudarenes, Catalonia Spain

**Keywords:** Feed intake, Growth, Intramuscular fat, Leptin, Meat quality, Swine, Animal breeding, Genetic association study, Genetic markers, Genotype, Heritable quantitative trait

## Abstract

The recessive T allele of the missense polymorphism rs709596309 C > T of the leptin receptor gene is associated with intramuscular fat. However, its overall impact on pork production is still partial. In this work, we investigated the all-round effects of the TT genotype on lean growth efficiency and carcass, meat and fat quality using data from an experiment that compared the performance of 48 TT and 48 C– (24 CT and 24 CC) Duroc barrows. The TT pigs were less efficient for lean growth than the C– pigs. Although heavier, their carcasses had less lean content, were shorter and had lighter loins. Apart from increasing marbling and saturated fatty acid content, changes caused by the TT genotype in meat and fat quality are likely not enough to be perceived by consumers. The effect on visual marbling score exceeded that on intramuscular fat content, which suggests a direct influence of the T allele on the pattern of fat distribution in muscle. With current low-protein diets, the T allele is expected to be cost-effective only in niche markets where a very high level of marbling is critical.

## Introduction

Leptin is a hormone secreted mainly by white adipocytes that regulates food intake and energy balance in mammals^1^. Although leptin deficiency leads to excessive food intake and increased body fat mass, obesity is mostly characterized not by leptin deficiency, but by hyperleptinemia. This is due to impaired leptin-mediated signaling that decreases tissue sensitivity to leptin^2^. Defective mutations in the leptin receptor gene (*LEPR*) are one of the causes for leptin resistance. In pigs, it has been shown that the homozygous for the recessive T allele of the missense polymorphism rs709596309 C > T of *LEPR*^3^ display higher levels of circulating leptin and fat deposition^4,5^, a clinical outcome that is compatible with a defective expression of the leptin receptor^6^.

The TT pigs not only deposit more subcutaneous and inner fat but also intramuscular fat (IMF), both in raw^5^ and dry-cured products^7^. It is generally accepted that IMF has a favorable effect on the sensory attributes of pork^8,9^, although its influence on acceptability depends on the consumer habits^10–12^ and the type of product. Thus, in pork products such as dry-cured ham, IMF content is closely connected to the consumers' preference^13^. Previous results have shown that TT pigs are heavier at weaning^14^ and at the end of fattening^15^ than C– (CC and CT) pigs. However, there is still limited experimental evidence on its impact on feed efficiency^16,17^. This poses a challenge to the use of this allele in pig breeding, as the potential benefits on meat quality need to be contrasted against the expected losses in lean growth efficiency and retail cut distribution. Therefore, the aim of this work was to investigate in a single experiment the effect of the *LEPR* rs709596309 C > T variant on lean growth efficiency during fattening and its relationship with carcass and meat quality traits.

## Materials and methods

### Experimental design and performance traits

The pigs used in this research were 96 barrows (48 TT and 48 C–, of which 24 were CT and 24 CC) from litters produced by 37 sows and 16 boars from the same Duroc line^18^. All pigs were previously genotyped for the *LEPR* rs709596309 C > T variant as described by Solé et al.^17^. At around 65 days of age (SD 2), pigs were delivered in one batch to the IRTA swine test station in Monells (Department of Climate Action, Food and Rural Agenda, Govern of Catalonia, Spain) for performance testing. There, the barrows were housed in 48 alternating pens of two pigs (1 m^2^/pig) with the same genotype (TT or C–) and reared under the same experimental conditions until 202 days (SD 2) of age. In order to mitigate potential maternal effects and competition at feeding, live body weight at the beginning of the test was equalized across genotypes (0.9 kg of difference) and within pen (0.3 kg of difference, on average). During the fattening period, all pigs had ad libitum access to commercial growing (until 90 days of age; 10.0 MJ/kg of net energy, 15.0% crude protein) and finishing (from 90 days of age to slaughter; 10.2 MJ/kg of net energy, 13.7% crude protein) diets (Table S1, Esporc, Riudarenes, Girona, Spain). At around 70 (69), 90 (92), 140 (142), 155 (155) and 200 (202) days of age, every pig was individually weighed and its backfat and loin thickness at 5 cm off the midline at the position of the last rib were measured using a portable ultrasonic scanner (Piglog 105; Frontmatec, Kolding, Denmark). Feed intake was recorded daily on a pen basis and summed up for each age interval. The average daily gain, feed intake and feed conversion ratio of the pen in each age interval were calculated thereafter. The residual feed intake of the pen was determined as the difference between the actual and the expected feed intake, which was estimated as the feed intake adjusted for the average metabolic body weight during the period plus the body weight and backfat thickness gain throughout the period^19^. The coefficient of determination for feed intake regression models ranged from 0.66 (from 140 to 155 days of age) to 0.80 (from 155 to 200 days of age).

### Carcass quality traits

All pigs were slaughtered in the same abattoir at 203 days of age (SD 2), where carcass weight was recorded, and carcass backfat and loin thickness were ultrasonically predicted with an automatic carcass grading equipment (AutoFOM, SFK-Technology, Denmark) at 6 cm off the midline between the third and fourth last ribs. The dressing percentage was calculated as the ratio of carcass weight and live body weight at 200 days while the carcass lean percentage was estimated in accordance with the prediction equation approved for Spain (Decision 2012/384/UE). After chilling at 2 °C for approximately 24 h, two additional measurements of carcass backfat thickness were directly taken using a ruler. The first was obtained at the level of the last rib, in the same location as the in vivo measurements, whereas the second one was recorded at the level of the gluteus medius muscle. Carcass length was measured from the anterior edge of the symphysis pubic to the recess of the first rib. Then, each carcass was divided into primary cuts and the hams, and the loin were individually weighted. Immediately after quartering, a section of around 250 g from the middle part of the longissimus muscle (LM) and from the gluteus medius muscle (GM), including subcutaneous fat, were taken. Both samples were individually vacuum-packed and transported during the same day to the laboratory.

### Cured ham processing

Left hams were individually traced and dry-cured through a four-step process^7^. First, hams were covered with salt and kept for 10 to 14 days at 2 ºC. Then, they were dried for 3 months following a slow ramp of increasing temperatures (from 3 to 10 ºC). After that, the hams were ripened for 6 months at 9 to 14 ºC. Finally, in the last ripening step, they were allocated into a single seasoning batch and kept in room temperature (15 to 20 ºC) for 16 months until around one third of the initial weight was lost. At the end of this process, the final weight of the bone-in cured ham and the weight loss during the whole dry-curing process were recorded. A slice from the middle of the ham including the biceps femoris (BF) and the semitendinosus (ST) muscles was taken, individually vacuum-packaged and then transported to the laboratory for further determinations.

### Meat and fat quality traits

Once in the laboratory, each LM and GM sample was split into two parts. One of the subsamples was used for immediate analysis while the other was stored in deep freeze until required. With the first subsample, pH and color readings were made on the exposed surface of LM and GM as well as color readings of the gluteal subcutaneous fat^7^. The pH was measured with a portable pH-meter (pH 7 Vio, XS-Instruments, Carpi, Italy) and color space coordinates^20^ for lightness (L*), redness (a*) and yellowness (b*) with a spectrophotometer (CM-700d, Konica Minolta Sensing Inc., Osaka, Japan). For each color coordinate, the final value was the average of six measurements taken in triplicate at two muscle sites. The Hue angle (h* = arctan(b*/a*), expressed in degrees; from dark to white) and chroma (C* = (a*^2^ + b*^2^)^0.5^; from dull to vivid intensity) variables were calculated. Moreover, a zenithal photograph of the exposed surface of LM was taken at 30 cm using a 12-megapixel camera with a wide-angle lens. Each photo was graded for marbling by 12 meat industry experts and 48 non-expert consumers according to the marbling standards of the National Pork Producers Council, USA (1: not at all, to 10: very much), and the result compared with the abattoir internal score for fat infiltration (from 1: little marbling, to 5: very much marbling), which was taken on the same surface of the raw LM by a technician of the cutting room at the moment of sampling. At least 20 g of each muscle was used to determine dry matter in duplicate by drying 24 h at 102 ºC in an air oven.

The second subsample was used for fat analysis. Defrosted muscle samples were trimmed of subcutaneous and intermuscular fat, freeze-dried and pulverized. Then, IMF content and fatty acid composition in LM and GM as well as fatty acid composition of the gluteal subcutaneous fat were determined in duplicate by quantitative determination of the individual fatty acids by gas chromatography^21^. The proportion of saturated fatty acids (SFA: C14:0, C16:0, C18:0, and C20:0); monounsaturated fatty acids (MUFA: C16:1n-9, C18:1n-7, C18:1n-9, and C20:1n-9); and polyunsaturated fatty acids (PUFA; 18:2n-6, C18:3n-3, C20:2n-6, and C20:4n-6) were expressed as percentages relative to total fatty acid content. The IMF content was calculated as the sum of each individual fatty acid expressed as triglyceride equivalents on a dry tissue basis^22^.

### Cured ham and fat quality traits

Dry-cured ham slices were placed on a polystyrene white tray. Color measurements were made directly on the exposed surface of BF and ST. Then, BF and ST muscles were dissected out from the ham slice and minced. Around 4 g of each muscle was used to determine dry matter while the rest of the sample was stored at − 20 ºC, freeze-dried and pulverized for fatty acid analysis. Color, dry matter and fatty acid composition were determined following the same procedures described above for raw LM and GM. Also, identically to LM and GM, a zenithal photograph of the exposed surface of the slice was taken. A panel of 16 consumers graded each slice for marbling according to the standards of the National Pork Producers Council, USA.

### Gene expression analysis

Differential gene expression of *LEPR* genotypes was analyzed using RNA‑seq data of 38 pigs (12 TT, 17 CT, and 9 CC) of the same Duroc line^23^. Total RNA was isolated from semimembranosus muscle samples using TRI Reagent (Invitrogen, Thermo Scientific, Waltham, MA, USA) and Direct-zol™ RNA Miniprep Plus Kit (Zymo Research, BioSystems, CA, USA) according to the manufacturer’s protocol. The RNA samples were sequenced by Centre Nacional d’Anàlisi Genòmica (CNAG-CRG, Barcelona, Spain, http://www.cnag.crg.eu/). Libraries were prepared using the TruSeq SBS v-3HS kit (Illumina, San Diego, CA) according to the manufacturer’s protocol. Each library was paired-end sequenced (2 × 100 bp) to 65 M reads with phred quality score 80–90% in a Hi-Seq 2000 platform. After the alignment with *Sus scrofa* reference genome (Sscrofa11.1), reads for each *LEPR* genotype were counted with the Feature Counts v1.24.1 software^24^.

### Statistical analyses

The effect of the *LEPR* genotype (TT and C–) on gene expression was estimated using a model that included the *LEPR* genotype of the individual as systematic effect. For individual performance, carcass and quality traits, the model also included the *LEPR* genotype of the dam (TT and C–) as systematic effect and the pig polygenic effect. In addition, age at measurement (for performance traits), carcass weight (for carcass and quality traits), and IMF (for fatty acid content) were added as covariates. In matrix notation, the model was **y** = **Xb** + **Za** + **e**, where **y** is the vector of observations for a trait; **b**,** a** and **e** are the vectors of systematic (pig and sow *LEPR* genotype and the corresponding covariate), polygenic and residual effects, respectively; and **X** and **Z** are the incidence matrices that relate **b** and **a** with **y**, respectively. Inferences were done in a Bayesian setting using the TM software^25^. The traits were assumed to be conditionally normally distributed as [**y** | **b**, **a**, **I**σ_e_^2^] ~ N (**Xb** + **Za**, **I**σ_e_^2^), where σ_e_^2^ is the residual variance and **I** the appropriate identity matrix. The pig effects conditional on the additive genetic variance σ_a_^2^ were assumed multivariate normally distributed with mean zero and variance **A**σ_a_^2^, where **A** was the numerator relationship matrix calculated from a two-generation pedigree. For feeding traits, records were taken on a pen basis, so the observations were the average of the two pigs in the pen. Accordingly, the pig polygenic effect was dropped from the model and the average age at measurement of the two pigs in the pen was used as a covariate. Marginal posterior distributions for all unknowns were estimated using Gibbs sampling^26^. Statistical inferences (namely, posterior means and SD, posterior probabilities of differences being greater or lower than 0 [P_0_], and the highest posterior density region at 95% [HPD95]) were derived from the samples of the marginal posterior distribution using a unique chain of 1,000,000 iterations, where the first 200,000 were discarded and 1 sample out of 1000 iterations was retained. In particular, the effect of the *LEPR* genotype was estimated as the mean of the marginal posterior distribution of the difference between genotypes. Convergence was tested using the *Z*-criterion of Geweke and visual inspection of convergence plots. We considered that there was very strong, strong or moderate evidence of difference between genotypes when P_0_ was at least 0.99, 0.95 or 0.90, respectively.

### Ethical approval

Pigs used in the study were raised and slaughtered following applicable regulations and good practice guidelines on the protection of animals kept for farming purposes, during transport and slaughter. In accordance with European Directive 2010/63/EU and Spanish Royal Decree 53/2013, no invasive procedures or treatments were performed on the animals in this study. The experimental protocol was reviewed by the Ethical Committee on Animal Experimentation of the University of Lleida (CEEA 04-06/21).

## Results

### Gene expression

**Figure 1:**
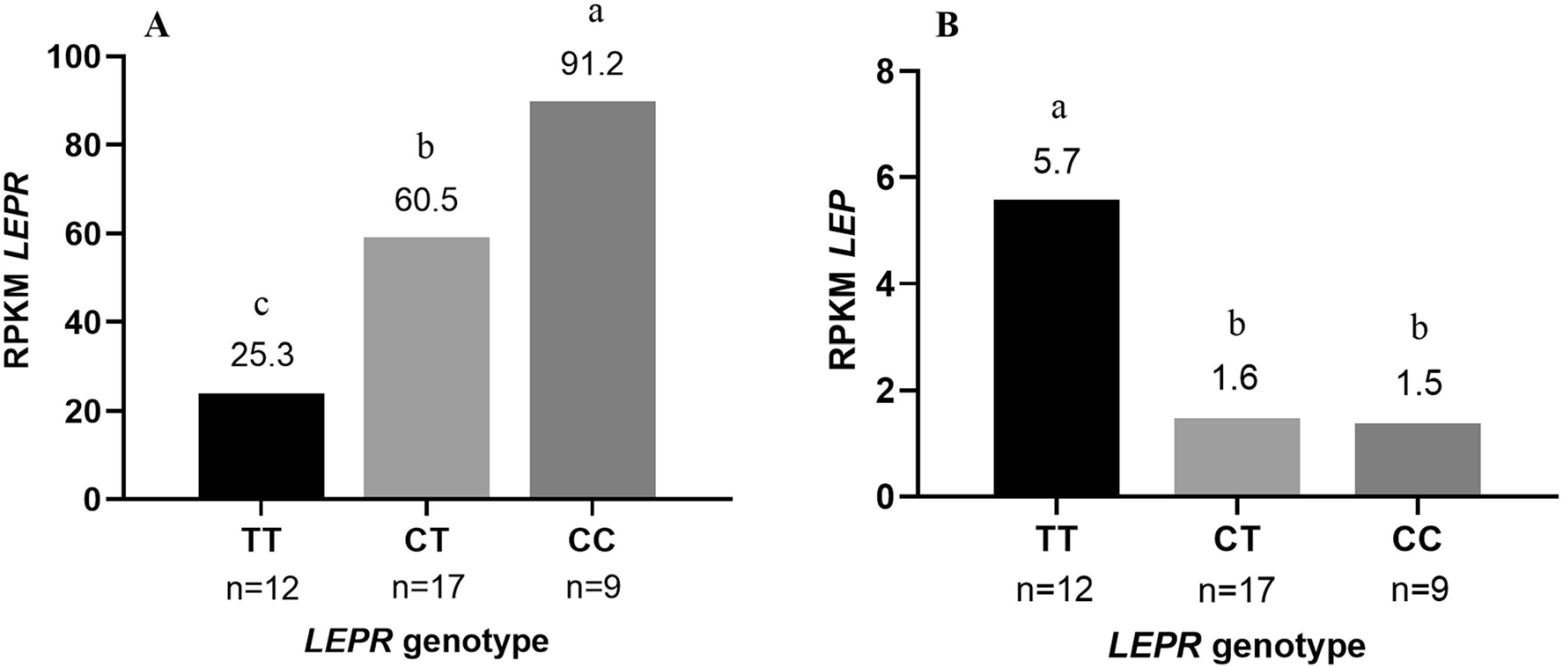
Average reads per kilobase per million mapped reads (RPKM) of (**A**) leptin receptor (*LEPR*) and (**B**) leptin (*LEP*) transcripts by *LEPR* genotype (TT, CT and CC).. Within transcript, means with different letters differ (P_0_ > 0.95). P_0_: Posterior probability of the difference between genotypes being greater (if positive) or lower (if negative) than zero. Leptin receptor expression was different between *LEPR* genotypes (P_0_ > 0.99, between TT and CT, and P_0_ = 0.98, between CT and CC) while leptin expression differed between TT and CT (P_0_ > 0.99), but not between CT and CC (P_0_ = 0.50).

Gene expression analysis was performed in the semimembranosus muscle, since it was not possible to obtain GM and LM samples immediately after slaughter. Leptin receptor and leptin gene expression in the semimembranosus muscle of the three *LEPR* genotypes is given in Fig. 1. As expected, the relative expression of *LEPR* was 2.8-fold higher (P_0_ > 0.99) in C– pigs than in TT pigs. In contrast, the relative expression of leptin was 3.8-fold higher (P_0_ > 0.99) in TT pigs as compared to C– pigs. The CC pigs had 1.5-fold higher (P_0_ = 0.98) expression of *LEPR* than CT pigs, but no difference was detected between them for leptin expression.

### Performance traits

On average, TT pigs were heavier and fatter than C– pigs throughout fattening (Table 1), with the maximum difference reached at the end of the period, from 155 to 200 days of age (+ 4.9 kg, P_0_ = 0.95, for live weight, and + 2.4 mm, P_0_ = 0.99, for backfat thickness). The negative effect of TT on loin thickness was not so evident (− 1.2 mm, P_0_ = 0.88, at the end of fattening). Although fatter, the TT pigs were able to grow faster during fattening (+ 36 g/d, P_0_ = 0.94). As shown in Table 2, the increased growth rate of the TT pigs was accompanied by an even larger increase of feed intake (+ 227 g/d, P_0_ > 0.99). As a result, the TT pigs had a higher feed conversion ratio during fattening than C– pigs (+ 124 g/kg, P_0_ > 0.99). However, from 155 to 200 days, feed conversion ratio was similar between both genotypes (− 12 g/kg, for TT, P_0_ = 0.52), with feed intake (+ 342 g/d, P_0_ > 0.99) more aligned with weight gain (+ 75 g/d, P_0_ = 0.93) between genotypes. The difference between genotypes became much lower and less evident for the residual feed intake (+ 45 g/d, P_0_ = 0.87, from 75 to 200 days), a result that indicates that around 80% of the excess feed consumed by the TT pigs can be attributed to differences in the level of production (i.e. size and body weight and fat gain).

**Table 1:** Raw mean and mean (SD) of the marginal posterior distribution of the difference between *LEPR* genotypes (TT - C–) for live weight and backfat and loin thickness at different ages (in days, d) during the fattening period.

Trait	Mean	Difference between genotypes			
		TT - C–	SD	P_0_^a^	HPD95^b^
Live body weight, kg					
70 d	22.9	1.3	0.8	0.95	− 0.2; 2.9
90 d	41.0	1.9	1.2	0.95	− 0.3; 4.3
140 d	79.8	2.6	1.8	0.92	− 1.0; 6.4
155 d	92.5	2.6	2.2	0.89	− 2.1; 6.6
200 d	133.8	4.9	3.1	0.95	− 1.0; 11.3
Backfat thickness, mm					
140 d	12.6	1.7	0.6	0.99	0.4; 2.9
155 d	14.8	2.3	0.8	> 0.99	0.8; 3.9
200 d	25.2	2.4	1.1	0.99	0.2; 4.4
Loin thickness, mm					
140 d	41.2	0.2	0.8	0.61	− 1.3; 1.7
155 d	42.7	1.2	1.2	0.85	− 1.1; 3.4
200 d	45.7	− 1.2	1.1	0.88	− 3.5; 0.9

^a^P_0_: Posterior probability of the difference between genotypes being greater (if positive) or lower (if negative) than zero. ^b^HPD95: highest posterior density region at 95%.

**Table 2:** Raw mean and mean (SD) of the marginal posterior distribution of the difference between *LEPR* genotypes (TT - C–) for feeding traits at different age intervals (in days, d) during the fattening period.

Trait	Mean	Difference between genotypes			
		TT - C–	SD	P_0_^a^	HPD95^b^
Daily feed intake, g					
70 d to 90 d	1595	178	65.2	> 0.99	56; 307
90 d to 140 d	2232	124	85.5	0.93	− 41; 288
140 d to 155 d	3106	265	200	0.91	− 140; 622
155 d to 200 d	3638	342	132	> 0.99	78; 586
70 d to 200 d	2704	227	74	> 0.99	83; 367
Average daily gain, g/d					
70 d to 90 d	787	34	32	0.85	− 26; 95
90 d to 140 d	774	4	36	0.55	− 66; 74
140 d to 155 d	977	2	77	0.52	− 135; 160
155 d to 200 d	879	75	50	0.93	− 25; 167
70 d to 200 d	833	36	23	0.94	− 8; 78
Residual feed intake, g/d					
70 d to 140 d	0	28	35	0.78	− 33; 101
140 d to 155 d	0	33	122	0.62	− 203; 252
155 d to 200 d	0	56	68	0.79	− 68; 193
70 d to 200 d	0	45	39	0.87	− 21; 127
Feed conversion ratio, g/kg					
70 d to 90 d	2026	131	52	> 0.99	31; 232
90 d to 140 d	2900	108	78	0.92	− 34; 263
140 d to 155 d	3230	243	181	0.91	− 86; 598
155 d to 200 d	4197	− 12	183	0.52	− 348; 355
70 d to 200 d	3248	124	55	> 0.99	28; 237

^a^P_0_: Posterior probability of the difference between genotypes being greater (if positive) or lower (if negative) than zero. ^b^HPD95: highest posterior density region at 95%.

### Carcass quality traits

The differences between the TT and C– pigs for carcass traits are given in Table 3. The TT pigs presented a lower dressing percentage (− 0.6%, P_0_ = 0.91) and, in line with on-live measurements, their carcasses were heavier (+ 4.1 kg, P_0_ > 0.99) but fatter (− 3.8% of lean percentage, P_0_ > 0.99). Moreover, the carcasses of the TT pigs were shorter (− 1.1 cm, P_0_ = 0.96) and had thinner (− 4.6 mm, P_0_ > 0.99) and lighter (− 0.3 kg, P_0_ > 0.99) loins. No sufficient evidence was found to support that the weight of hams was lighter in TT pigs (− 0.2 kg, P_0_ = 0.76). Once adjusted for carcass weight, the weight of the dry-cured ham of TT pigs (− 0.0 kg, P_0_ = 0.53) and the weight loss during the dry-curing process (− 0.3 kg, P_0_ = 0.69) also did not differ between genotypes.

**Table 3:** Raw mean and mean (SD) of the marginal posterior distribution of the difference between *LEPR* genotypes (TT - C–) for carcass traits.

Trait^c^	Mean	Difference between genotypes			
		TT - C–	SD	P_0_^a^	HPD95^b^
Carcass weight, kg	100.2	4.1	2.6	0.94	− 0.9; 9.2
Dressing percentage, %	74.9	− 0.6	0.4	0.91	− 1.4; 0.3
Carcass length, cm	89.0	− 1.1	0.7	0.96	− 2.3; 0.3
Weight of hams, kg	24.6	− 0.2	0.2	0.76	− 0.7; 0.3
Weight of loin, kg	5.5	− 0.3	0.1	> 0.99	− 0.5; − 0.2
Backfat thickness at 3–4 rib, mm	35.2	3.4	1.4	0.99	0.8; 6.6
Backfat thickness at last rib, mm	26.4	2.9	0.9	> 0.99	1.2; 4.7
Backfat thickness at gluteus, mm	27.4	2.2	1.1	0.98	− 0.0; 4.1
Loin thickness at 3–4 rib, mm	39.5	− 4.6	1.7	> 0.99	− 7.7; − 1.1
Lean percentage, %	37.7	− 3.8	1.5	> 0.99	− 6.6; − 0.9

^a^P_0_: Posterior probability of the difference between genotypes being greater (if positive) or lower (if negative) than zero. ^b^HPD95: highest posterior density region at 95%. ^c^Values adjusted for carcass weight.

### Muscle quality traits

The *LEPR* genotype had little influence on muscle color and pH (Tables 4 and 5). Taking the results of raw LM and GM as a whole, they indicate that the TT genotype had a positive impact on L* (+ 1.8, P_0_ = 0.93, in GM) and pH (+ 0.1, P_0_ = 0.98, in LM), while negative on a* (− 0.6, P_0_ = 0.96, in GM). Although similar in magnitude, the TT genotype increased a* in cured ST (+ 0.6, P_0_ = 0.95) and b* in cured BF (+ 0.8, P_0_ = 0.93). The effect of the TT genotype on IMF was more evident in the cured (+ 1.3%, P_0_ > 0.99, in BF) than in the raw muscles, where it was only moderately evident in GM (+ 0.8%, P_0_ = 0.91). In contrast, both industry experts and consumers were able to detect a clear effect of the TT genotype on fat infiltration (+ 0.5, P_0_ > 0.99) and marbling, both in raw loin (+ 0.8, P_0_ = 0.99, and + 0.6, P_0_ = 0.96, for experts and consumers, respectively) and in dry-cured ham (+ 1.2, P_0_ > 0.99). There was a positive correlation of IMF in LM with visual fat infiltration (0.53, P_0_ > 0.99) and marbling (0.64 and 0.63, P_0_ > 0.99, for experts and consumers, respectively) and between fat infiltration and marbling (0.56 and 0.55, P_0_ > 0.99, for experts and consumers, respectively). The correlation of marbling in dry-cured ham with IMF was also positive but higher than in loin (0.80 and 0.76, P_0_ > 0.99, for BF and ST, respectively).

**Table 4:** Raw mean and mean (SD) of the marginal posterior distribution of the difference between *LEPR* genotypes (TT - C–) for quality traits in raw longissimus and gluteus medius muscles.

Trait^c^	Longissimus muscle					Gluteus medius muscle				
	Mean	TT—C–	SD	P_0_^a^	HPD95^b^	Mean	TT - C–	SD	P_0_^a^	HPD95^b^
L*	49.1	0.9	0.8	0.86	− 0.6; 2.4	48.8	1.8	1.2	0.93	− 0.5; 4.3
a*	1.3	− 0.3	0.3	0.90	− 0.9; 0.2	3.3	− 0.6	0.3	0.96	− 1.2; 0.1
b*	12.1	− 0.1	0.3	0.59	− 0.7; 0.5	8.7	0.1	0.5	0.58	− 0.9; 1.0
C*	12.2	− 0.1	0.3	0.61	− 0.8; 0.5	9.4	− 0.2	0.5	0.63	− 1.1; 0.7
h*	61.4	− 12.5	14.3	0.82	− 40.6; 14.9	69.1	3.3	2.1	0.95	− 0.9; 7.3
pH at 24 h	5.7	0.1	0.0	0.98	0.0; 0.2	6.2	0.5	0.7	0.77	− 0.8; 1.9
Dry matter, %	29.4	− 0.3	0.6	0.72	− 1.5; 0.7	32.1	1.0	0.8	0.88	− 0.7; 2.5
IMF, %	7.6	0.0	0.6	0.55	− 1.1; 1.1	8.1	0.8	0.6	0.91	− 0.3; 1.9
Fat infiltration	2.7	0.5	0.2	> 0.99	0.0; 1.4	–	–	–	–	–
Marbling-E	4.9	0.8	0.4	0.99	0.1; 1.6	–	–	–	–	–
Marbling-NE	5.5	0.6	0.4	0.96	− 0.0; 1.3					

^a^P_0_: Posterior probability of the difference between genotypes being greater (if positive) or lower (if negative) than zero. ^b^HPD95: highest posterior density region at 95%; ^c^L*: Lightness; a*: redness; b*: yellowness; C*: chroma; h*: hue angle; pH at 24: pH at 24 h postmortem; *IMF* intramuscular fat on a wet-matter basis; Fat infiltration: marbling graded on raw loin from 1 (low) to 5 (high) according to the customary abattoir standards; Marbling-E (Marbling-NE): marbling graded on photo from 1 (low) to 10 (high) by 12 industry experts (E) and 48 non-expert consumers (NE) according to the standards of the National Pork Producers Council, USA. Values adjusted for carcass weight.

**Table 5:** Raw mean and mean (SD) of the marginal posterior distribution of the difference between *LEPR* genotypes (TT - C–) for quality traits in dry-cured biceps femoris and semitendinosus muscles.

Trait^c^	Biceps femoris muscle					Semitendinosus muscle				
	Mean	TT - C–	SD	P_0_^a^	HPD95^b^	Mean	TT - C–	SD	P_0_^a^	HPD95^b^
L*	44.0	0.3	0.7	0.66	− 1.0; 1.7	44.8	− 0.2	0.8	0.58	− 1.7; 1.4
a*	12.8	0.2	0.5	0.67	− 0.6; 1.2	12.4	0.6	0.4	0.95	− 0.1; 1.3
b*	4.5	0.8	0.5	0.93	− 0.2; 1.8	6.0	0.1	0.4	0.63	− 0.6; 1.0
C*	13.7	0.5	0.5	0.83	− 0.4; 1.6	14.0	0.6	0.4	0.96	− 0.1; 1.2
h*	18.8	3.1	2.1	0.94	− 0.8; 7.0	25.2	− 0.5	1.8	0.60	− 3.7; 3.2
Dry matter, %	49.0	0.8	0.6	0.89	− 0.3; 2.0	55.5	0.1	0.9	0.51	− 1.6; 1.8
IMF, %	7.7	1.3	0.5	> 0.99	0.4; 2.2	14.6	0.7	0.9	0.76	− 1.1; 2.4
Marbling-NE	5.6	1.2	0.3	> 0.99	0.5; 1.8					

^a^P_0_: Posterior probability of the difference between genotypes being greater (if positive) or lower (if negative) than zero. ^b^HPD95: highest posterior density region at 95%; ^c^L*: Lightness; a*: redness; b*: yellowness; C*: chroma; h*: hue angle; IMF: intramuscular fat on a wet-matter basis; Marbling-NE: marbling graded on photo from 1 (low) to 10 (high) by 16 non-expert consumers according to the standards of the National Pork Producers Council, USA. Values adjusted for carcass weight.

### Fat quality traits

The fatty acid composition of IMF in LM and GM is shown in Table 6 and of IMF in BF and ST in Table 7. The IMF of the TT pigs presented higher SFA in all muscles, either raw (+ 1.0% and + 1.3%, for LM and GM, respectively, P_0_ > 0.99) or cured (+ 0.6%, P_0_ = 0.98, for BF, and + 0.7%, P_0_ = 0.97. for ST). The increase in SFA was mostly at the expense of PUFA (− 0.9% and − 1.1%, P_0_ > 0.99, for LM and GM, respectively, and − 0.4%, P_0_ = 0.97, for BF), excepting for ST, where MUFA was the one that decreased in favor of SFA (− 0.7%, P_0_ = 0.99). In fact, the MUFA content was relatively stable for LM, (− 0.2%, P_0_ = 0.70), GM (− 0.2%, P_0_ = 0.82) and BF (− 0.3%, P_0_ = 0.80), but not for ST (− 0.7%, P_0_ = 0.99). This was because, unlike in other muscles, C18:1n-9 in ST experienced a considerable decrease in TT pigs (− 0.5%, P_0_ = 0.97). As in muscle, the *LEPR* genotype had a minor influence on subcutaneous fat color (Table S2), which also revealed a more saturated fatty acid composition (Table S3). Thus, the subcutaneous fat of the TT pigs had greater L* (+ 0.8, P_0_ = 0.98) and lower a* (− 0.3, P_0_ = 0.99) and b* (− 0.9, P_0_ > 0.99). The main change in the subcutaneous fatty composition caused by the TT genotype was a substitution of SFA for MUFA (+ 0.6%, P_0_ = 0.99), which was mainly due to a decrease in C18:1n-9 (− 0.7%, P_0_ = 0.98).

**Table 6:** Raw mean and mean (SD) of the marginal posterior distribution of the difference between *LEPR* genotypes (TT - C–) for fatty acid composition in longissimus and gluteus medius muscles.

Fatty acid^c^, % FA	Longissimus muscle					Gluteus muscle				
	Mean	TT - C–	SD	P_0_^a^	HPD95	Mean	TT - C–	SD	P_0_^a^	HPD95^b^
SFA	39.8	1.0	0.3	> 0.99	0.5; 1.6	38.2	1.3	0.3	> 0.99	0.7, 2.0
C14:0	1.6	0.0	0.0	0.71	− 0.1; 0.0	1.5	0.0	0.0	0.56	− 0.1, 0.0
C16:0	26.6	0.5	0.2	> 0.99	0.2; 0.9	25.5	0.7	0.2	> 0.99	0.3, 1.1
C18:0	11.5	0.6	0.2	> 0.99	0.2; 1.0	11.1	0.6	0.2	> 0.99	0.3, 0.9
C20:0 (× 10)	1.9	0.1	0.0	> 0.99	0.0; 0.2	1.4	0.1	0.0	0.99	0.0, 0.2
MUFA	52.9	− 0.2	0.3	0.70	− 0.7; 0.5	52.6	− 0.2	0.3	0.82	− 0.8, 0.3
C16:1n-9	3.8	− 0.1	0.1	0.89	− 0.3, 0.1	3.6	− 0.1	0.1	0.70	− 0.2, 0.1
C18:1n-9	43.9	0.1	0.3	0.59	− 0.5; 0.6	43.9	− 0.2	0.3	0.77	− 0.6, 0.4
C18:1n-7	4.4	− 0.1	0.1	0.99	− 0.3; − 0.0	4.3	− 0.1	0.1	0.96	− 0.2, 0.0
C20:1n-9 (× 10)	8.5	0.3	0.2	0.94	− 0.0; 0.7	8.4	0.3	0.2	0.93	− 0.0, 0.7
PUFA	7.3	− 0.9	0.2	> 0.99	− 1.3; − 0.5	9.1	− 1.1	0.3	> 0.99	− 1.6, − 0.5
C18:2n-6	5.9	− 0.7	0.2	> 0.99	− 1.1; − 0.4	7.4	− 0.9	0.2	> 0.99	− 1.3, − 0.4
C18:3n-3 (× 10)	3.1	− 0.5	0.1	> 0.99	− 0.7, − 0.2	4.3	− 0.6	0.2	> 0.99	− 0.9, − 0.3
C20:2n-6 (× 10)	3.0	− 0.3	0.1	> 0.99	− 0.5, − 0.1	3.9	0.3	0.1	> 0.99	− 0.6, − 0.1
C20:4n-6 (× 10)	7.7	− 0.6	0.2	> 0.99	− 1.1, − 0.1	9.1	− 0.9	0.4	0.99	− 1.8, − 0.1
SFA/MUFA (× 10)	7.5	0.2	0.9	> 0.99	0.1, 0.4	7.3	0.3	0.9	> 0.99	0.1, 0.4
SFA/PUFA	5.7	0.9	0.2	> 0.99	0.5; 1.2	4.3	0.7	0.2	> 0.99	0.4, 1.1
MUFA/PUFA	7.5	0.9	0.2	> 0.99	0.5; 1.3	5.9	0.7	0.2	> 0.99	0.3, 1.2
n6/n3	22.4	0.8	0.3	> 0.99	0.2; 1.4	20.1	0.3	0.4	0.77	− 0.5, 1.1

^a^P_0_: Posterior probability of the difference between genotypes being greater (if positive) or lower (if negative) than zero. ^b^HPD95: highest posterior density region at 95%; ^c^*SFA* saturated fatty acids (C14:0 + C16:0 + C18:0 + C20:0); *MUFA* monounsaturated fatty acids (C16:1n-9 + C18:1n-9 + C18:1n-7 + C20:1n-9); *PUFA* polyunsaturated fatty acids (C18:2n-6 + C18:3n-3 + C20:2n-6 + C20:4n-6); n6: C18:2n-6 + C20:2n-6 + C20:4n-6; and n3: C18:3n-3. Values adjusted for intramuscular fat content.

**Table 7:** Raw mean and mean (SD) of the marginal posterior distribution of the difference between *LEPR* genotypes (TT - C–) for meat quality traits and fatty acid composition in biceps femoris and semitendinosus muscles.

Fatty acid^c^, % FA	Biceps femoris muscle					Semitendinosus muscle				
	Mean	TT - C–	SD	P_0_^a^	HPD95	Mean	TT - C–	SD	P_0_^a^	HPD95^b^
SFA	36.8	0.6	0.3	0.98	0.0; 1.2	39.0	0.7	0.3	0.97	0.1; 1.3
C14:0	1.4	0.0	0.0	0.29	− 0.1; 0.0	1.4	0.0	0.0	0.61	-0.1; 0.0
C16:0	24.7	0.2	0.2	0.90	− 0.1; 0.6	25.8	0.1	0.2	0.78	-0.3; 0.5
C18:0	10.6	0.4	0.2	> 0.99	0.1; 0.7	11.6	0.6	0.2	> 0.99	0.2; 0.9
C20:0 (× 10)	0.2	0.0	0.2	0.53	− 0.3; 0.3	0.2	0.1	0.0	0.94	0.0; 0.1
MUFA	55.0	− 0.3	0.3	0.80	− 0.9; 0.3	54.7	− 0.7	0.3	0.99	− 1.2; − 0.1
C16:1n-9	3.9	− 0.1	0.1	0.90	− 0.3; 0.1	3.4	− 0.1	0.1	0.89	− 0.3; 0.1
C18:1n-9	45.5	0.0	0.3	0.56	− 0.6; 0.5	46.1	− 0.5	0.3	0.96	− 1.1; 0.0
C18:1n-7	5.0	− 0.1	0.1	0.95	− 0.2; 0.0	4.3	− 0.1	0.1	0.97	− 0.2; 0.0
C20:1n − 9 (× 10)	0.8	0.5	0.2	> 0.99	0.2; 0.9	0.9	0.6	0.3	> 0.99	0.1; 1.2
PUFA	8.2	− 0.4	0.2	0.97	− 0.9; 0.0	6.3	0.0	0.3	0.54	− 0.5; 0.5
C18:2n-6	6.6	− 0.4	0.2	0.99	− 0.8; − 0.1	5.4	− 0.1	0.2	0.68	− 0.5; 0.3
C18:3n-3 (× 10)	0.3	− 0.2	0.1	0.97	− 0.4; 0.0	0.3	0.0	0.1	0.45	− 0.3; 0.3
C20:2n-6 (× 10)	0.3	− 0.0	0.1	0.40	− 0.4; 0.0	0.3	0.1	0.1	0.77	− 0.1; 0.4
C20:4n-6 (× 10)	1.0	0.1	0.5	0.61	− 0.8; 1.0	0.3	0.5	0.4	0.89	− 0.3; 1.4
SFA/MUFA (× 10)	7.0	1.4	0.9	0.93	− 3.0; 3.0	7.1	2.0	0.9	0.99	0.2; 3.6
SFA/PUFA	4.6	1.0	0.2	0.99	0.1; 0.6	6.3	0.3	0.3	0.87	− 0.2; 0.7
MUFA/PUFA	6.9	0.3	0.2	0.94	− 0.1; 0.7	8.9	− 0.9	0.3	> 0.99	− 1.5; − 0.3
n6/n3	26.4	0.4	0.6	0.75	− 0.7; 1.4	24.2	0.3	0.5	0.72	− 0.7; 1.3

^a^P_0_: Posterior probability of the difference between genotypes being greater (if positive) or lower (if negative) than zero. ^b^HPD95: highest posterior density region at 95; ^c^*SFA* saturated fatty acids (C14:0 + C16:0 + C18:0 + C20:0); *MUFA* monounsaturated fatty acids (C16:1n-9 + C18:1n-9 + C18:1n-7 + C20:1n-9); *PUFA*: polyunsaturated fatty acids (C18:2n-6 + C18:3n-3 + C20:2n-6 + C20:4n-6); n6: C18:2n-6 + C20:2n-6 + C20:4n-6; and n3: C18:3n-3. Values adjusted for intramuscular fat content.

## Discussion

This study reports the results from an experiment designed to investigate the effects of the *LEPR* rs709596309 C > T variant on lean growth efficiency and carcass, meat and fat quality traits in pigs. Although other variants have been reported in the *LEPR* gene^3,14,27–29^, this is the one that presents the most consistent results across studies. The T allele involves the amino acid change *L663F* in the coded protein^3^ that reduces leptin receptor functionality^6^. Leptin is produced by adipocytes and acts as an anorexigenic signal in hypothalamic neurons, so loss of leptin signaling causes hyperphagia and decreased energy expenditure^30^. Óvilo et al.^6^ found that TT pigs had a third of *LEPR* hypothalamic expression compared to CC pigs, with CT pigs showing intermediate values. The reduced expression of *LEPR* in the hypothalamus of the pigs carrying the T allele is in line with the results found here in muscle, thus contributing to support causality and the paracrine role of leptin receptors in maintaining local tissue homeostasis^31,32^. As expected in the leptin-resistance obesity model, the deficit in *LEPR* expression was counteracted by increased leptin expression. However, this compensatory effect occurred in TT pigs, but not in CT pigs, suggesting that leptin resistance only appears when a minimum threshold of LEPR is not reached, a hypothesis that, in turn, would explain the recessive behavior of the T allele.

Pigs used here were from a Duroc line that produces for premium markets. Unlike lines used as terminal sires of 3-way crosses^33,34^, the Duroc lines used as purebred or 2-way Duroc crossbreds have been selected less aggressively for lean growth to keep IMF at required levels^35^, such as those needed to produce dry-cured ham, where IMF is highly appreciated^36^. Therefore, alleles associated with fatness, such as the T allele, are more likely to be present in traditional Duroc lines^4,5^ and in local breeds. For instance, the T allele is almost fixed in Iberian and related breeds^14,37^.

We previously demonstrated that TT sows provide a negative maternal environment to piglets that reduces their weight at weaning^15^, which is counteracted by a positive direct effect of the TT genotype on the weight and vitality of the piglets at birth as well as on growth during lactation^14^. As a continuation of this research, in the present study we further pursued on the effect of the TT genotype on performance traits during the growing-fattening period. Our findings showed that TT pigs were heavier than C– pigs at slaughter, corroborating prior results in this Duroc line using on-farm data^17^ and from two backcrosses involving Iberian, Duroc and Landrace pigs^6^. The TT pigs also had higher feed intake throughout fattening, but it was only during the finishing period (155 to 200 days) that increased feed intake had a correlative response in growth rate. In controlled research settings, with high energy-dense diets and low stocking densities, such as this one, feed intake should be close to or just at the maximum potential for voluntary energy intake. At this point, pigs are no longer in an energy-dependent state of growth^38^. Results indicate that TT pigs have a greater capacity for energy intake and so for growth, which is consistent with a thrifty behavior^17^. On the other hand, as long as reduced protein diets affect growth performance^39^, then, conversely, leaner C– pigs should be more prone to further decline in growth.

The TT pigs grew faster but had a poorer feed conversion ratio. The difference between genotypes for feed conversion decreased with age until vanishing in the finishing period. In terms of weight gain, the TT pigs are less efficient at younger ages because they initiate fat mass expansion earlier, but, with time, the fat-to-lean deposition ratio becomes more balanced across genotypes and so also the feed conversion ratio. This trend is likely to be more pronounced here, since our pigs were barrows, which are known to start to deposit fat earlier and are fatter than entire males^40^. Feed efficiency, however, did not differ between genotypes when it was measured in terms of residual feed intake, i.e. if also fat accretion is considered. Thus, in global terms, TT pigs are not intrinsically worse feed converters than C– pigs, but rather metabolically different, in the sense that their dietary intake is directed more towards lipid than protein accumulation to optimize energy savings. In this way, despite being heavier, TT carcasses had less lean mass (− 4.7 kg, P_0_ > 0.99, and − 3.8 kg, P_0_ > 0.99, unadjusted and adjusted for carcass weight, respectively, and calculated as the product of carcass weight and lean percentage). The storage of excess fat in TT pigs decreased dressing percentage and carcass quality and led to a lighter loin than C– pigs. Each carcass was divided into standardized commercial cuts according to customary procedure used in Spanish abattoirs. We were able to individually track untrimmed hams (whole leg) and loin, but not other economically relevant cuts, which were weighted collectively by *LEPR* genotype. In accordance with the observed trends for individual results, TT pigs, as compared with C– pigs, showed a lower proportion of hams (25.0% vs 25.8%), loin (5.4% vs 5.7%), shoulder (whole leg, 14.7% vs 15.2%) and neck filet (4.8% vs 5.0%) in the carcass, while higher proportion of belly (9.8% vs. 8.8%), as expected for a fatty cut^41^.

Increased fatness in TT carcasses was not accompanied by a marked positive response in IMF, as we have previously observed throughout fattening^42^ and at slaughter^5^, and as could be anticipated by the positive correlation of IMF with backfat thickness in this Duroc line^43^, including the one found here (+ 0.29, P_0_ = 0.99). Although IMF tended to be higher in TT pigs, we were only able to detect this effect in cured BF, the least fatty among the four investigated muscles, where therefore differential leptin sensitivity between *LEPR* genotypes is expected to be more acute. Several hormones, including leptin, regulate fatty acid metabolism in skeletal muscle^44,45^. Leptin reduces intramuscular triglycerides by increasing fatty acid oxidation and triglyceride hydrolysis while reducing fatty acid esterification. Leptin resistance downregulate these processes in skeletal muscle of obese individuals^46^ and can be differentially expressed depending on gender^47^ and muscle^48^. Our experimental pigs achieved an extreme level of IMF compared to present-day standards, showing twice as much IMF as their commercial counterparts^49^, so we can surmise that, except for BF, they were at a stage in which adipocyte hypertrophy was close to a plateau. In addition to effective response to selective breeding^35^, this elevated IMF level is explained by research experimental conditions, including the diet. Current diets are at least 3% lower in protein than previous ones^5^ and reduced protein diets increase IMF^39,50^. This, in combination with high nutrient intake during growth, could have increased the number and size of adipocytes in skeletal muscle^51,52^ and blurred the effect of *LEPR* on IMF, particularly in LM, GM and ST. Leptin gene expression differs across adipose depots, marking especially subcutaneous fat^53^. Intramuscular adipocytes are less metabolically active and less sensible to leptin as compared with subcutaneous and perirenal adipocytes. Findings in pigs would confirm this to the extent that circulating leptin is more correlated with backfat thickness and intermuscular fat than with IMF^54^.

In contrast to IMF, the TT pigs evidenced a higher level of visible fat than C– pigs, both in loin and dry-cured ham. Fat infiltration and marbling scores are two correlated measures of visible fat. Fat perception depends on IMF but also on how it is distributed over the exposed surface of the muscle. The correlation of IMF in LM with fat infiltration and marbling was positive but moderate, thereby suggesting that, in the TT loins, IMF was displayed in a more striking appearance, probably because it is assembled according to a more perceptible density distribution. Human subjectivity and fatty acid composition can lead to misreading fat. Since SFA have higher melting points, at room temperature fat with more SFA becomes more visible. These potential perturbations did not neutralize the positive effect of the TT genotype on fat infiltration and marbling, which was maintained when extreme ratings were discarded or after adjusting the scores for IMF, SFA or PUFA. Marbling is a key cue of visual appearance and visual appearance is highly related with consumers' expectations of meat quality. Although some marbling is generally appreciated, a feeling of excess fat, coupled with increased SFA, can be counterproductive and may deter more diet- and health-conscious consumers from choosing TT pork at the point of purchase.

Besides IMF and visual appearance, pH, color, and fatty acid composition are the other main sources of sensory differentiation in pork^55–57^. The most remarkable effect of *LEPR* on these traits was on fatty acid composition, particularly increasing SFA. It is known that SFA increases with fat content, both in IMF and subcutaneous fat^58^. However, TT pigs showed a greater proportion of SFA even adjusting for IMF or backfat thickness, which confirms that the endogenous synthesis of fat begins earlier in TT pigs than in C– pigs. Increased SFA is achieved at the expense of PUFA or MUFA, depending on the amount of adipose tissue. In general, SFA replace PUFA when the fat content is lower (i.e. in LM, GM, and BF) or MUFA when the fat content is higher (i.e. in ST and subcutaneous fat). Given that there were only minor differences between genotypes for pH and moisture, the positive effect of the TT genotype on L* (and negative on a*) should be attributed to increased visual fat and SFA content^59^. Previous studies have reported that IMF increases lightness^60^ and decreases the presence of pigments such as myoglobin^61^. The effects on color were more evident in subcutaneous fat, where b* was also affected. Unlike dry-cured ham^7^, raw subcutaneous fat of TT pigs was less yellow, which is consistent with the fact that yellowness is associated with lower solid fat content and with processing time^62^.

## Conclusions

This study provides further evidence that leptin plays an important role in the regulation of feed intake and development of adiposity. Findings here demonstrate that the TT pigs are less efficient at producing pork and have worse carcass quality. Apart from increasing marbling, changes in meat quality caused by the TT genotype are minor and likely not enough to be perceived by consumers. The effect on visual marbling exceeds IMF, which argues for a specific effect of *LEPR* on the IMF distribution pattern. At least in fat pigs fed low-protein diets, the benefits of the T allele are, at best, limited to satisfying niche markets aimed at consumers who are willing to pay for a high level of marbling. On the contrary, the T allele provides a convenient model for research into dietary intake, metabolism and adiposity.

### Supplementary Information

Supplementary Table S1.

Supplementary Table S2.

Supplementary Table S3.

## Data Availability

The data generated or analyzed during this study are included in this published article or are available from the corresponding author on reasonable request.

## Abbreviations

a*: Color space coordinate for redness
b*: Color space coordinate for yellowness
BF: Biceps femoris muscle
GM: Gluteus muscle
HPD95: Highest posterior density interval at 95% of probability
IMF: Intramuscular fat
L*: Color space coordinate for lightness
*LEPR*: Leptin receptor gene
LM: Longissimus muscle
MUFA: Monounsaturated fatty acids
P_0_: Posterior probability of a difference being greater or lower than 0
PUFA: Polyunsaturated fatty acids
SD: Standard deviation
ST: Semitendinosus muscle
SFA: Saturated fatty acids
