# Supplementary material for: Impact of the leptin receptor gene on pig performance and quality traits
Source: Sci Rep. 2024 May 9;14:10652. doi: 10.1038/s41598-024-61509-1 (PMC11087582; doi:10.1038/s41598-024-61509-1)
Supplement: Supplementary file 1 — Supplementary Table S1. [file 41598_2024_61509_MOESM1_ESM.docx]

**Table S1.** Composition and nutrients of the diets fed during the fattening period

|  | From 30 to 90 days (growing) | From 90 to 200 days (finishing) |
| --- | --- | --- |
| Ingredients, % as-fed basis |  |  |
| Corn | 35.00 | - |
| Barley | 34.30 | 35.00 |
| Wheat | - | 20.09 |
| Soybean meal | 14.79 | 8.27 |
| Rye | 3.00 | 11.80 |
| Wheat bran | - | 7.00 |
| Pea | 5.68 | 4.00 |
| Fat | 1.45 | 4.83 |
| Beet Pulp | - | 4.00 |
| Triticale | 2.00 | - |
| Rapeseed | - | 1.50 |
| CaCO_3_ | 0.62 | 1.00 |
| CaHPO_4_ | 0.67 | - |
| L-Lysine 50% | 0.49 | 0.44 |
| NaCl | 0.45 | 0.44 |
| Nutrients, % |  |  |
| Net energy, MJ/Kg | 10.00 | 10.25 |
| Crude protein | 15.00 | 13.70 |
| Neutral detergent fibre | 12.65 | 15.83 |
| Crude fat | 3.50 | 6.27 |
| Ashes | 4.71 | 4.56 |
| Total calcium | 0.75 | 0.70 |
| Total phosphorous | 0.46 | 0.41 |
| Digestible phosphorus | 0.32 | 0.26 |
| SID Leucine | 1.042 | 0.770 |
| SID Lysine | 0.875 | 0.735 |
| SID Valine | 0.608 | 0.525 |
| SID Threonine | 0.577 | 0.478 |
| SID Isoleucine | 0.527 | 0.442 |
| SID Methionine | 0.288 | 0.220 |
| SID Cysteine | 0.234 | 0.220 |
| SID Tryptophan | 0.158 | 0.147 |
| C16:0, Palmitic | 0.54 | 1.09 |
| C18:0, Stearic | 0.23 | 0.40 |
| C16:1, Palmitoleic | 0.01 | 0.13 |
| C18:1, Oleic | 0.94 | 2.20 |
| C18:2, Linoleic | 1.02 | 1.23 |
