# Supplementary material for: Impact of the leptin receptor gene on pig performance and quality traits
Source: Sci Rep. 2024 May 9;14:10652. doi: 10.1038/s41598-024-61509-1 (PMC11087582; doi:10.1038/s41598-024-61509-1)
Supplement: Supplementary file 2 — Supplementary Table S2. [file 41598_2024_61509_MOESM2_ESM.docx]

**Table S2**. Raw mean and mean (SD) of the marginal posterior distribution of the difference between *LEPR* genotypes (TT - C−) for color traits in subcutaneous fat.

| Trait ^c^ |  | Difference between genotypes | | | | |
| --- | --- | --- | --- | --- | --- | --- |
|  | Mean | TT - C– | | SD | P_0_ ^a^ | HPD95 ^b^ |
| L* | 77.6 | 0.8 | 0.4 | | 0.98 | 0.1; 1.5 |
| a* | -0.3 | -0.3 | 0.1 | | 0.99 | -0.6; -0.0 |
| b* | 9.3 | -0.9 | 0.3 | | >0.99 | -1.5; -0.3 |
| C* | 9.3 | -0.9 | 0.3 | | >0.99 | -1.5; -0.3 |
| h* | -28.6 | -46.3 | 20.6 | | 0.99 | -89.7; -9.4 |

^a^ P_0_: Posterior probability of the difference between genotypes being greater (if positive) or lower (if negative) than zero. ^b^ HPD95: highest posterior density region at 95%; ^c^ L*: Lightness; a*: redness; b*: yellowness; C*: chroma; and h*: hue angle. Values adjusted for carcass weight.
