# Supplementary material for: Impact of the leptin receptor gene on pig performance and quality traits
Source: Sci Rep. 2024 May 9;14:10652. doi: 10.1038/s41598-024-61509-1 (PMC11087582; doi:10.1038/s41598-024-61509-1)
Supplement: Supplementary file 3 — Supplementary Table S3. [file 41598_2024_61509_MOESM3_ESM.docx]

**Table S3.** Raw mean and mean (SD) of the marginal posterior distribution of the difference between *LEPR* genotypes (TT - C−) for dry matter and fatty acid composition in subcutaneous fat.

| Trait ^c^ |  | | Difference between genotypes | | | |
| --- | --- | --- | --- | --- | --- | --- |
|  | Mean | | TT - C- | SD | P_0_ ^a^ | HPD95 ^b^ |
| Dry Matter, % |  | 94.24 | 0.9 | 0.4 | >0.99 | 0.2; 1.6 |
| Fatty acid, % FA |  |  |  |  |  |  |
| SFA |  | 39.3 | 0.6 | 0.4 | 0.95 | -0.1; 1.4 |
| C14:0 |  | 1.3 | -0.0 | 0.0 | 0.92 | -0.1; 0.0 |
| C16:0 |  | 24.6 | -0.9 | 0.3 | >0.99 | -1.5; -0.3 |
| C18:0 |  | 13.2 | 0.7 | 0.3 | 0.99 | 0.2; 1.3 |
| C20:0 (x10) |  | 0.2 | 0.0 | 0.1 | 0.67 | -0.1; 0.1 |
| MUFA |  | 48.8 | -0.8 | 0.3 | 0.99 | -1.4; -0.1 |
| C16:1n-9 |  | 1.9 | -0.1 | 0.1 | 0.97 | -0.2; 0.0 |
| C18:1n-9 |  | 42.8 | -0.7 | 0.3 | 0.98 | -1.2; 0.0 |
| C18:1n-7 |  | 3.0 | -0.1 | 0.1 | 0.99 | -0.2; 0.0 |
| C20:1n-9 (x10) |  | 1.2 | 0.4 | 0.3 | 0.92 | -0.2; 1.1 |
| PUFA |  | 11.9 | 0.2 | 0.3 | 0.79 | -0.3; 0.7 |
| C18:2n-6 |  | 10.3 | 0.2 | 0.2 | 0.78 | -0.2; 0.6 |
| C18:3n-3 (x10) |  | 0.7 | 0.1 | 0.2 | 0.77 | -0.2; 0.4 |
| C20:2n-6 (x10) |  | 0.7 | 0.2 | 0.2 | 0.77 | -0.1; 0.5 |
| C20:4n-6 (x10) |  | 0.2 | -0.1 | 0.1 | 0.84 | -0.2; 0.0 |
| SFA/MUFA (x10) |  | 8.1 | 2.6 | 1.3 | 0.98 | 0.3; 5.4 |
| SFA/PUFA |  | 3.3 | 0.0 | 0.1 | 0.68 | -0.1; 0.2 |
| MUFA/PUFA |  | 4.1 | -0.1 | 0.1 | 0.86 | -0.3; 0.1 |
| n6/n3 |  | 16.6 | -0.1 | 0.1 | 0.66 | -0.3; 0.2 |

^a^ P_0_: Posterior probability of the difference between genotypes being greater (if positive) or lower (if negative) than zero. ^b^ HPD95: highest posterior density region at 95%; ^c^ SFA: saturated fatty acids (C14:0+C16:0+C18:0+C20:0); MUFA: monounsaturated fatty acids (C16:1n-9+C18:1n-9+ C18:1n-7+ C20:1n-9); PUFA: polyunsaturated fatty acids (C18:2n-6 + C18:3n-3 + C20:2n-6 + C20:4n-6); n6: C18:2n-6+ C20:2n-6 + C20:4n-6; and n3: C18:3n-3. Values adjusted for carcass weight (dry matter) and backfat thickness at gluteus (fatty acids).
